# The Prevalence of Duplicate Prescription of Oral Antibiotic Drugs in Outpatient Care among People Insured by Corporate Health Insurance Societies in Japan

**DOI:** 10.3390/healthcare12111150

**Published:** 2024-06-05

**Authors:** Kenichi Fujimoto, Shinichi Tanihara

**Affiliations:** 1 Department of Public Health, School of Medicine, Kurume University, Kurume-City 830-0011, Japan; fujimotokenichi@hotmail.co.jp; 2 Eisai Co., Ltd., Tokyo 112-8088, Japan

**Keywords:** antibiotics, duplicate prescription, outpatients, Japan, health insurance

## Abstract

Inappropriate antimicrobial use is a global problem, especially because the use of antimicrobials in excess of appropriate doses is associated with increased antimicrobial resistance. Duplicate prescriptions are an issue contributing to inappropriate antimicrobial use. This study aimed to analyse antibiotic prescriptions during a specific month to examine the frequency of outpatients receiving duplicate antibiotic prescriptions and the associated determinants. Utilizing the Japan Medical Data Centre health insurance claim database, we retrospectively identified 527,110 insured individuals with at least one medicine prescription in October 2014. Data regarding age, gender, antibiotic drug usage, and health insurance status were extracted. Duplicate prescriptions entailed a patient receiving two or more prescriptions of systemic antibiotics from multiple facilities within one month. The risk factors for duplicate antibiotic prescriptions were evaluated using logistic regression analysis. Of the total sample, 131,709 individuals (25.0%) received antibiotics, and 24,529 of these individuals (18.6%) had duplicate prescriptions. Third-generation cephalosporins accounted for the largest proportion of prescriptions (37.4%). Duplicate prescriptions were significantly associated with sex, age, medical facilities, and health insurance status. These findings could help to identify patients at risk of duplicate antibiotic prescriptions, highlighting the need to promote proper antimicrobial use in both patients and medical professionals.

## 1. Introduction

The inappropriate use of antimicrobial drugs, such as inappropriate prescription and overprescription, has been reported to be associated with an increased risk of microbes developing antimicrobial resistance (AMR) [1]; therefore, measures to promote the appropriate antimicrobial stewardship are being considered worldwide [2,3,4]. Inappropriate antimicrobial use is a global issue, especially because the use of antimicrobials in excess of appropriate doses is associated with increased healthcare costs and the development of AMR [4,5].

Potentially inappropriate prescriptions include duplicate prescriptions in which a patient is prescribed drugs of the same class by more than one healthcare provider. Duplicate prescriptions exhibit a significant correlation with prescription frequency, particularly manifesting a greater prevalence of duplicated medications within the 0–19 years age group [6]. Notably, cough and cold drugs, as well as antibacterial drugs, have exhibited a higher possibility of duplication [7]. Furthermore, antihypertensive drugs are the most frequently prescribed medications among individuals aged 65 years and older [7]. A study conducted at a university hospital reported that duplicate prescribing of antimicrobials occurred at 6% [8], and a study conducted in South Korea using health insurance claim data found a 7.6% rate of occurrence [9].

In Japan, the utilisation of third-generation cephalosporins, quinolones, and macrolides is higher than that in other countries [10]. Consequently, the Japanese government had set a target to reduce the use of these drugs by 50% before 2020 [11]. Although most antimicrobials are prescribed to outpatients [12], few studies have examined the duplicate prescriptions of antimicrobials they receive.

Therefore, this study aims to analyse antibiotic prescriptions during a specific month using a large-scale health insurance claim (HIC) database and examine the frequency of outpatients who received duplicate antibiotic prescriptions and the factors associated with duplicate prescriptions.

## 2. Methods

### 2.1. Setting

There are three major types of health insurance systems operate in Japan: National Health Insurance (NHI), Employee Health Insurance (EHI), and the Late-Stage Elderly Medical Care System (LTC) [13]. NHI is a system in which municipalities establish insurance for their residents. Self-employed, unemployed, or retired individuals aged <75 years are enrolled in the NHI. EHI is a system for employees and their dependents and is divided into two major categories: the Corporate Health Insurance Society (CHIS) and the Japan Health Insurance Association (JHIA). The CHIS is a health insurance society established by relatively large enterprises to benefit employees. The JHIA is a health insurance system for companies with relatively few employees. The LTC is a system for older adults aged 75 years or older. 

### 2.2. Data

This cross-sectional study was conducted in October 2014 using data from the Japan Medical Data Centre (JMDC) claims database. The JMDC contains outpatient and inpatient HICs data and eligibility information from several CHISs (comprising 1,582,156 insured and dependent individuals as of April 2014). The database includes information on insurance status, sex, inpatient and outpatient visits, diagnoses, prescriptions, medical facilities, and subscriber eligibility information [14], and includes dependent individuals <75 years.

### 2.3. Variables

Drugs are coded according to the Anatomical Therapeutic Chemical Classification based on the Chemical Classification System. Outpatients with at least one antibiotic prescription were identified using claims from October 2014. All the prescribed antibiotic drugs were aggregated for patients with multiple claims.

Duplicate prescriptions are defined as two or more prescriptions of systemic antibiotics (J01) belonging to the same class from two or more facilities within one month [7]. These include cases where the first and second antibiotic classes differ. 

The majority of cephalosporins used in Japan are third-generation cephalosporins. The following ATC codes were also used from previous studies: penicillin (J01C), first-generation cephalosporins (J01DB), second-generation cephalosporins (J01DC), third-generation cephalosporins (J01DD), macrolides (J01FA), quinolones (J01M), sulphonamides and trimethoprim (J01E), tetracyclines (J01A), and other antibiotics (J01B: amphenicols, J01DH: carbapenems, J01DI: other cephalosporins and penems, J01FF: lincosamides, J01G: aminoglycoside antibacterials, and J01X: other antibacterials) [15].

The status of antibiotic prescriptions and the proportion of duplicate prescriptions were calculated by age group and antibiotic drug class.

We examined the patients who were prescribed antibiotic medications and checked for duplicate prescriptions according to sex and age group (0–9, 10–19, 20–29, 30–39, 40–49, 50–59, 60–69, 70–74 years). We also described the classes of multiple antibiotic prescriptions in each age group and analysed the classes and proportions of antibiotics prescribed.

### 2.4. Analyses 

Logistic regression analysis was conducted to investigate the factors associated with duplicate antibiotic prescriptions. The variables used in the multivariate analysis were derived from previous studies on variables related to antibiotic prescriptions [6,15,16]. The variables included: age (0–4 years, 5–9 years, 10–19 years, 20–64 years, and <65 years), sex (female, male), facility scale (hospital: >20 beds, clinic: less than 20 beds), and insurance status (dependent or insured). The dummy variables indicated age, sex, facility scale, and insurance status as potential confounders. Facility scale handling was classified into two patterns: hospital visits (including cases in which the patient visited a hospital and clinic in the same month) and clinic-only visits (cases in which the patient visited a clinic only).

Statistical significance was indicated by a *p*-value < 0.05, and a 95% confidence interval (CI) was used for the association of these variables with duplicate prescriptions. The statistical analysis was performed using SAS version 9.4.

### 2.5. Ethical Considerations

The original HIC data contained identifiers, such as names and personal identification numbers, for insured individuals and their dependents. These were converted into anonymised IDs using hash functions in the JMDC claims database. No correspondence table between the data could identify the insured persons and the anonymised ID. Furthermore, as only the anonymised database was provided to the researcher, no information that could identify individuals was accessible. This study was approved by the Kurume University Ethical Review Committee on Medical Care (study number 22258).

## 3. Results

As of October 2014, 527,110 insured people had at least one pharmacy claim in the database, and 131,709 (25.0%) were prescribed at least one antibiotic drug. The mean age of patients prescribed antibiotics was 23.6 years (standard deviation (SD) ± 19.5), and 49.3% of them were men.

The number of outpatients who met the definition of having a duplicate prescription was 24,529 (18.6%) (Table 1). 

The mean age of patients with duplicate prescriptions was 22.0 years (SD ± 20.8), and 49.3% of them were men. The largest age group class was 70–74 years (26.5%), followed by 0–9 years (23.5%) and 60–69 years (23.0%) (Table 1).

The number of patients with duplicate prescriptions according to number of drug classes was 12,868 (52.8%) for one, 7449 (30.5%) for two, 3059 (12.5%) for three, 879 (3.6%) for four, and 138 (0.6%) for five or more classes. The highest proportion of prescriptions in all age groups was for one class of drugs and exceeded 50%. The proportion of prescriptions decreased in order from two or more classes (Table 2).

The most frequently prescribed antibiotics were third-generation cephalosporins (37.4%), followed by macrolides (25.9%) and quinolones (10.2%) (Table 3). Among those eligible for duplicate prescriptions, third-generation cephalosporins (27.4%) were the most commonly prescribed, followed by macrolides (21.1%) and quinolones (9.8%). The proportion of other antibiotic prescriptions increased with the number of duplicate prescriptions (Table 4).

Logistic regression analysis showed that the odds ratio (OR) for duplicate antibiotic prescriptions was higher for the 0–4 years age group compared to over 65 years age group (adjusted OR, 1.08; 95% CI, 1.01 to 1.17). By age group, 10–19 years had the lowest OR (adjusted OR, 0.57; 95% CI, 0.52 to 0.61). Regarding sex, females had a lower OR for duplicative prescriptions (adjusted OR, 0.96; 95% CI, 0.94 to 0.98). Regarding facility, hospitals had a higher OR than did clinics (adjusted OR, 1.81; 95% CI, 1.76 to 1.86). Furthermore, OR for insurance status was higher for dependents than for the main member (adjusted OR, 1.24; 95% CI, 1.19 to 1.28) (Table 5).

## 4. Discussion

Our analysis clarified the status of antibiotic prescriptions and duplicate prescriptions in Japan. Antibiotics were prescribed to 25% of insured individuals. Approximately 19% of the patients prescribed antibiotic drugs were classified as having duplicate prescriptions. Of these patients, nearly half utilised two or more antibiotic prescriptions. Third-generation cephalosporins were the most commonly prescribed antibiotics. The factors contributing to duplicate prescriptions were age, sex, medical facilities, and health insurance status. The proportion of duplicate prescriptions of antimicrobials was higher than that reported in international studies. However, due to differences in the definition of duplicate prescribing, general comparisons cannot be made.

In a previous study on antibiotic prescribing using NHI data from 2012 [15], in all age groups, 34.8% of prescriptions were for third-generation cephalosporins (J01D), 31.6% were for macrolides (J01F), 21.3% were for quinolones (J01M), and 4.8% were for penicillin (J01C). Compared to the present study’s results for patients eligible for duplicate prescriptions, these figures were higher for third-generation cephalosporins and quinolones (7.4% and 11.5%, respectively) and lower for penicillin (4.9%). These differences may reflect the characteristics of patients with duplicate prescriptions.

A possible reason for the higher OR in the 0–4 years age group is the higher likelihood that this age group would visit a physician if they had a cold. This may have resulted in the prescription of antibiotics in conjunction with physician visits. For the other age groups, this result may indicate a lower frequency of consultations due to colds as compared to the older adult age group. 

In the present study, females had a significantly lower OR for duplicate prescriptions. Regarding sex differences in the frequency of antibiotic prescribing, it has been reported that males are more likely to be prescribed with antibiotics than are females [15,16,17]. 

The results of our study are similar to those of previous studies. However, a study conducted in the United States reported a higher proportion of antibiotic prescriptions among women [18]. Therefore, future studies on sex differences in duplicate antibiotic prescriptions, including insurance schemes and access to healthcare, are needed for further investigation across cultures and countries.

In this study, patients visiting hospitals had a higher risk of receiving duplicate prescriptions than did those visiting clinics. This study examined antibiotic prescriptions within a specific month. It did not differentiate between patients who received antibiotic prescriptions at a clinic and were subsequently referred to a hospital where they received additional antibiotic prescriptions within the same month and those who received antibiotic prescriptions on the same day from multiple medical facilities. Consequently, this analysis included both appropriate and inappropriate antibiotic prescription practices; however, the inclusion of both practices was not anticipated to impact the analysis outcomes. Previous studies have indicated that first-time prescriptions of antibiotics are frequently prescribed in clinics [15,17]. It is assumed that antibiotics were prescribed in the clinic at the first visit, and when the patient’s symptoms did not improve, a second or subsequent visit was made to the hospital where more antibiotics were prescribed. Alternatively, the patient may have been severely ill and required multiple prescriptions [3]. Duplicate visits and multiple prescriptions in patients who visit multiple healthcare facilities can lead to overdosing and increase the risk of drug-resistant bacteria. This study did not include data on the prescription date, making it impossible to examine the sequence of duplicate visits. A more detailed analysis of visits to different medical facilities in the same month for the same injury or illness should be conducted in future research.

The findings of a previous study on antibiotic prescriptions for preschool children mostly matched the proportions of third-generation cephalosporins, macrolides, and penicillin in this study [16]. Furthermore, similar trends have been identified in other prior investigations [19]. Studies on antibiotic prescriptions have reported that clinics (adjusted OR 1.88), hospitals with fewer than 200 beds (adjusted OR 1.17), and smaller healthcare facilities are more likely to prescribe antibiotics for gastrointestinal infections [15]. These previous results suggest that clinics prescribe more antibiotics than do hospitals. In addition, 56% of antibiotic prescriptions are used for infections that have few indications [20]; therefore, examining the status of antibiotic prescriptions according to the injury or disease is a topic for future research. The percentage of other antibiotics, which are frequently prescribed for intestinal infections and administered orally or intravenously, was high at approximately 22%. Investigating the usage patterns of these other antibiotics represents another important area for future research. Furthermore, it has been reported that the frequency of duplicate prescriptions may be influenced by seasonal variations [7]. A study using one month of data from December 2012 reported more duplicate prescriptions for symptoms of colds and respiratory illnesses, whereas a study using April 2002 data reported high levels of duplicate prescriptions for allergy medications [6]. Going forward, the actual situation of duplicate prescriptions of antibiotics, considering seasonality, and the knowledge required to optimise the misuse and overuse of antibiotics must be clarified. 

This study used HIC data from several health insurance associations and considered visits to multiple medical facilities, which cannot be ascertained in an analysis of patients who visited a specific medical institution. Furthermore, by using eligibility information, this study’s analysis distinguished between the insured individuals and their dependents. The status of medical institution visits has been reported to differ depending on the type of health insurance coverage [21]. The system-specific antibiotic prescriptions obtained in a previous study [15] and the present study, both of which included individuals insured by the conventional NHI, comprised a high proportion for third-generation cephalosporins, macrolides, and quinolones. Since the medical treatment covered by the health insurance system in Japan is not dependent on the type of medical insurance, the results of this study demonstrate that antibiotic prescription status is also not dependent on the health insurance system, as supported by real-world data.

Our study has several limitations. First, it only analysed the antibiotic prescription status and did not consider individuals’ injuries or diseases. Assuming that the necessary antimicrobials are selected according to the injury or illness, the results of this study may reflect the actual disease diagnoses that occur in clinical practice. However, several previous studies have reported that antimicrobials are not always appropriately prescribed in outpatient clinics [16,17].

Second, duplicate prescriptions in this study were defined as multiple antibiotic prescriptions within one month. Therefore, it includes cases where antibiotics were prescribed based on clinical symptoms at the initial visit and subsequently changed based on test results, such as pathogen culture results. It also includes cases where several strains of antibiotics are prescribed simultaneously, such as for Helicobacter pylori eradication therapy. Therefore, the definition of duplicate prescriptions used in this study does not necessarily correspond to inappropriate antibiotic prescriptions. 

Third, the study used a database of health insurance associations, which includes few people aged 65 years and over and no people covered by the LTC system. Therefore, this study cannot be generalised to the broader Japanese population. Although the occurrence of injuries and illnesses is also influenced by age group, the results of this study, considering age groups, did not differ significantly from those of a previous study on the NHI-insured population [15]. This study reflects the current situation of antibiotic prescriptions in Japan for patients aged <75 years. 

Fourth, this study used HIC data from a specific one-month period and did not capture the actual situation of long-term antibiotic prescriptions. The duplication of antibiotic prescriptions may have persisted beyond the second month; however, comprehending such duplication over several months remains a challenge for future research. The data presented here reflect the situation as it was in 2014. Therefore, new data must be used in future research to examine the course of problems identified in this data analysis. It is believed that certain limitations mentioned above do not exert influence on the interpretation of the findings.

## 5. Conclusions

This study revealed that third-generation cephalosporins were prescribed at the highest rate among Japanese outpatients during October 2014. Among the antibiotic prescriptions, 18.6% were duplicate antibiotics prescribed within the one-month study period. Factors associated with duplicate prescriptions were age (0–4 years), sex (male), facility (hospital), and insurance status (dependent). Examining the actual situation of inappropriate antibiotic prescriptions considering these factors will enable the formulation of measures for the appropriate use of antimicrobials.

## Figures and Tables

**Table 1:** Number of patients prescribed antibiotics and percentage of patients eligible for duplicate prescriptions according to age group.

Age Group (Years) *	Number of Insured and Dependent (A)	Patients with Antibiotic Prescriptions (B, B/A (%))	Number of Patients with Duplicate Prescriptions (C, C/B (%))
0–9	117,459	47,603 (40.5)	11,189 (23.5)
10–19	59,638	18,978 (31.8)	2528 (13.3)
20–29	47,703	12,941 (27.1)	1715 (13.3)
30–39	73,958	19,139 (25.9)	2936 (15.3)
40–49	95,598	17,100 (17.9)	2724 (15.6)
50–59	82,913	10,414 (12.6)	2135 (20.5)
60–69	42,230	4708 (11.1)	1083 (23.0)
70–74	7611	826 (10.9)	219 (26.5)
Total	527,110	131,709 (25.0)	24,529 (18.6)
Age average (SD)	32.2 (20.9)	23.6 (19.5)	22.0 (20.8)
Sex (male, %)	51.8	49.3	49.3

* Employee Health Insurance does not include people aged 75 years or older.

**Table 2:** Number of patients with duplicate prescriptions and number of antibiotic classes used by age group during October 2014.

Age Group (Years) *	Number of Antibiotic Classes (*n*, %)					
	1	2	3	4	≥5	Total
0–9	5718	2440	982	278	65	9483 (38.9)
10–19	1385	531	194	41	6	2157 (8.8)
20–29	948	288	102	34	10	1382 (5.7)
30–39	1417	611	232	54	17	2331 (9.6)
40–49	1423	529	199	37	20	2208 (9.1)
50–59	1217	385	109	39	9	1759 (7.2)
60–69	632	198	44	24	10	908 (3.7)
70–74	128	42	4	2	1	177 (0.7)
Total	12,868 (52.8)	7449 (30.5)	3059 (12.5)	879 (3.6)	138 (0.6)	24,393 (100)

* Employee Health Insurance does not include people aged 75 years or older.

**Table 3:** Frequency of antibiotic prescriptions according to antibiotic class by age group.

Age Group (Years) †	Antibiotic Groups * (*n*, %)									
	Penicillin	First-Generation Cephalosporins	Second-Generation Cephalosporins	Third-Generation Cephalosporins	Macrolides	Quinolones	Sulfonamides and Trimethoprim	Tetracyclines	Other Antibiotics	All Antibiotics
0–9	7296	438	461	19,467	12,363	1400	217	238	5717	47,597 (36.2)
10–19	895	182	259	7640	5996	1169	108	854	1869	18,972 (14.4)
20–29	768	262	358	4640	2949	2036	107	815	990	12,925 (9.8)
30–39	1215	394	515	6717	4762	3045	133	590	1744	19,115 (14.5)
40–49	899	357	472	5657	4302	3041	208	485	1653	17,074 (13)
50–59	564	309	336	3448	2393	1789	220	247	1078	10,384 (7.9)
60–69	269	184	165	1441	1112	800	127	102	482	4682 (3.6)
70–74	42	30	30	247	193	148	19	17	98	824 (0.6)
Total	11,948 (9.1)	2156 (1.6)	2596 (2.0)	49,257 (37.4)	34,070 (25.9)	13,428 (10.2)	1139 (0.9)	3348 (2.5)	13,631 (10.4)	131,573 (100)

* Penicillin, J01C; first-generation cephalosporins, J01DB; second-generation cephalosporins, J01DC; third-generation cephalosporins, J01DD; macrolides, J01FA; quinolones, J01M; sulphonamides and trimethoprim, J01E; tetracyclines, J01A; other antibiotics, J01B (amphenicols), J01DH (carbapenems), J01DI (other cephalosporins and penems), J01FF (lincosamides), J01G (aminoglycoside antibacterials), and J01X (other antibacterials). † Employee Health Insurance does not include people aged 75 years or older.

**Table 4:** Frequency of antibiotic prescriptions by age group according to antibiotic class in duplicate prescriptions.

Age Group (Years) †	Antibiotic Groups * (*n*, %)									
	Penicillin	First-Generation Cephalosporins	Second-Generation Cephalosporins	Third-Generation Cephalosporins	Macrolides	Quinolones	Sulfonamides and Trimethoprim	Tetracyclines	Other Antibiotics	All Antibiotics
0–9	2760	193	213	5844	4662	960	145	107	4424	19,308 (46.3)
10–19	178	113	99	1139	1052	264	72	205	1023	4145 (9.9)
20–29	219	178	159	759	437	414	58	132	515	2871 (6.9)
30–39	369	258	314	1302	914	787	68	114	1066	5192 (12.4)
40–49	224	248	230	1135	835	769	90	120	1003	4654 (11.1)
50–59	200	220	209	793	580	573	90	92	728	3485 (8.3)
60–69	99	149	98	384	260	278	67	39	361	1735 (4.2)
70–74	18	27	17	83	57	57	14	10	67	350 (0.8)
Total	4067 (9.7)	1386 (3.3)	1339 (3.2)	11,439 (27.4)	8797 (21.1)	4102 (9.8)	604 (1.4)	819 (2.0)	9187 (22.0)	41,740 (100)

* Penicillin, J01C; first-generation cephalosporins, J01DB; second-generation cephalosporins, J01DC; third-generation cephalosporins, J01DD; macrolides, J01FA; quinolones, J01M; sulphonamides and trimethoprim, J01E; tetracyclines, J01A; other antibiotics, J01B (amphenicols), J01DH (carbapenems), J01DI (other cephalosporins and penems), J01FF (lincosamides), J01G (aminoglycoside antibacterials), and J01X (other antibacterials). † Employee Health Insurance does not include people aged 75 years or older.

**Table 5:** Risk factors and odds ratios of duplicate prescriptions.

Risk Factors	Antibiotic Prescription, *N* (%)	Unadjusted OR (95% CI)	Adjusted OR (95% CI)
Age (years) †			
0–4	29,261 (22.2)	1.02 (0.95 to 1.10)	1.08 (1.01 to 1.17) *
5–9	18,342 (13.9)	0.77 (0.71 to 0.84) **	0.83 (0.77 to 0.90) **
10–19	18,978 (14.4)	0.52 (0.49 to 0.57) **	0.57 (0.52 to 0.61) **
20–64	62,864 (47.7)	0.64 (0.60 to 0.69) **	0.74 (0.69 to 0.80) **
65–74	2264 (1.7)	1 (reference)	1 (reference)
Sex			
Female	64,889 (49.3)	1.00 (0.97 to 1.02)	0.96 (0.94 to 0.98) **
Male	66,820 (50.7)	1 (reference)	1 (reference)
Facility ^‡^			
Hospital	13,798 (10.5)	1.75 (1.70 to1.80) **	1.81 (1.76 to 1.86) **
Clinic	117,911 (89.5)	1 (reference)	1 (reference)
Insurance status			
Dependent	90,128 (68.4)	1.32 (1.28 to 1.35) **	1.24 (1.19 to 1.28) **
Insured	41,581 (31.6)	1 (reference)	1 (reference)

* *p* < 0.05, ** *p* < 0. 001. † Employee Health Insurance does not include people aged 75 years or older. ^‡^ Facilities classified as hospitals have over 20 beds, and those classified as clinics have less than 20 beds.

## Data Availability

Data are not publicly available due to data provider reasons.
